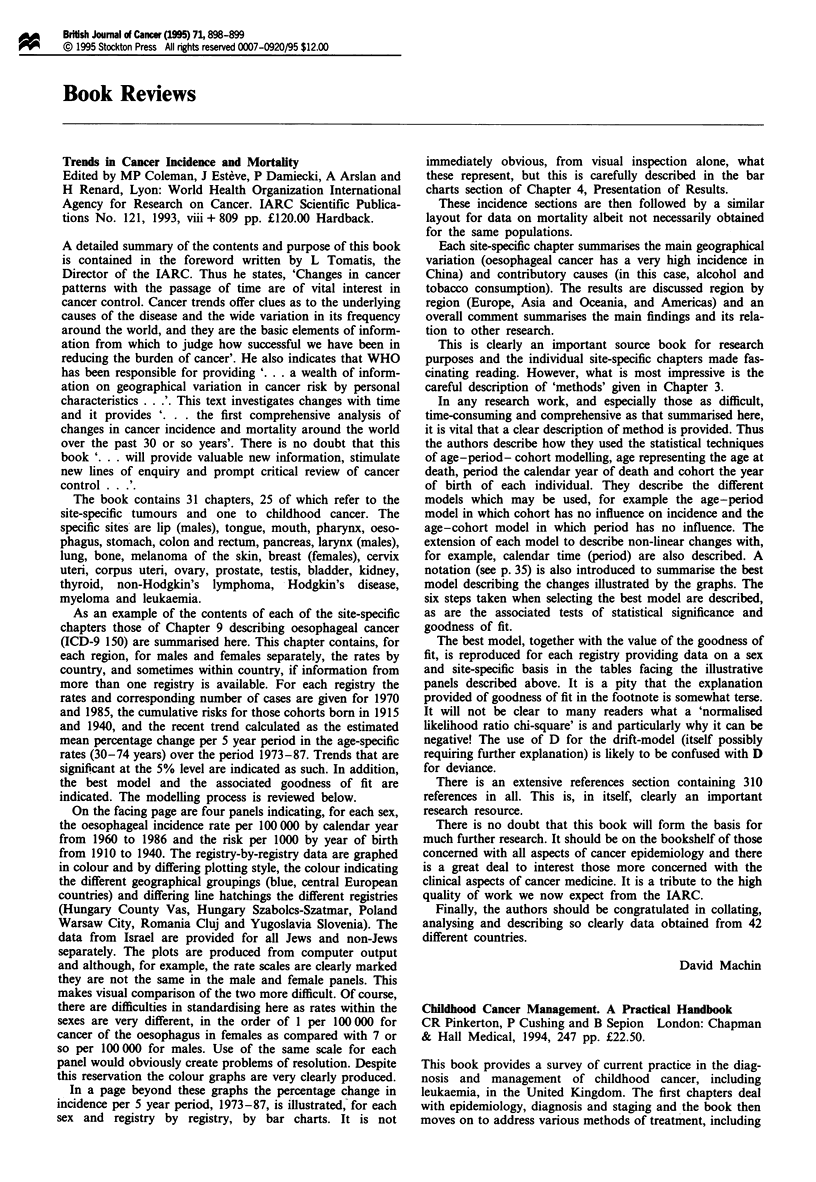# Trends in Cancer Incidence and Mortality

**Published:** 1995-04

**Authors:** David Machin


British Journal of Cancer (1995) 71, 898–899

© 1995 Stockton Press All rights reserved 0007–0920/95 $12.00

### Book Reviews

**Trends in Cancer Incidence and Mortality**
Edited by MP Coleman, J Estève, P Damiecki, A Arslan and H Renard, Lyon: World Health Organization International Agency for Research on Cancer. IARC Scientific Publications No. 121, 1993, viii + 809 pp. £120.00 Hardback.

A detailed summary of the contents and purpose of this book is contained in the foreword written by L Tomatis, the Director of the IARC. Thus he states, 'Changes in cancer patterns with the passage of time are of vital interest in cancer control. Cancer trends offer clues as to the underlying causes of the disease and the wide variation in its frequency around the world, and they are the basic elements of information from which to judge how successful we have been in reducing the burden of cancer'. He also indicates that WHO has been responsible for providing '. . . a wealth of information on geographical variation in cancer risk by personal characteristics . . .'. This text investigates changes with time and it provides '. . . the first comprehensive analysis of changes in cancer incidence and mortality around the world over the past 30 or so years'. There is no doubt that this book '. . . will provide valuable new information, stimulate new lines of enquiry and prompt critical review of cancer control . . .'.

The book contains 31 chapters, 25 of which refer to the site-specific tumours and one to childhood cancer. The specific sites are lip (males), tongue, mouth, pharynx, oesophagus, stomach, colon and rectum, pancreas, larynx (males), lung, bone, melanoma of the skin, breast (females), cervix uteri, corpus uteri, ovary, prostate, testis, bladder, kidney, thyroid, non-Hodgkin's lymphoma, Hodgkin's disease, myeloma and leukaemia.

As an example of the contents of each of the site-specific chapters those of Chapter 9 describing oesophageal cancer (ICD-9 150) are summarised here. This chapter contains, for each region, for males and females separately, the rates by country, and sometimes within country, if information from more than one registry is available. For each registry the rates and corresponding number of cases are given for 1970 and 1985, the cumulative risks for those cohorts born in 1915 and 1940, and the recent trend calculated as the estimated mean percentage change per 5 year period in the age-specific rates (30–74 years) over the period 1973–87. Trends that are significant at the 5% level are indicated as such. In addition, the best model and the associated goodness of fit are indicated. The modelling process is reviewed below.

On the facing page are four panels indicating, for each sex, the oesophageal incidence rate per 100 000 by calendar year from 1960 to 1986 and the risk per 1000 by year of birth from 1910 to 1940. The registry-by-registry data are graphed in colour and by differing plotting style, the colour indicating the different geographical groupings (blue, central European countries) and differing line hatchings the different registries (Hungary County Vas, Hungary Szabolcs-Szatmar, Poland Warsaw City, Romania Cluj and Yugoslavia Slovenia). The data from Israel are provided for all Jews and non-Jews separately. The plots are produced from computer output and although, for example, the rate scales are clearly marked they are not the same in the male and female panels. This makes visual comparison of the two more difficult. Of course, there are difficulties in standardising here as rates within the sexes are very different, in the order of 1 per 100 000 for cancer of the oesophagus in females as compared with 7 or so per 100 000 for males. Use of the same scale for each panel would obviously create problems of resolution. Despite this reservation the colour graphs are very clearly produced.

In a page beyond these graphs the percentage change in incidence per 5 year period, 1973–87, is illustrated, for each sex and registry by registry, by bar charts. It is not immediately obvious, from visual inspection alone, what these represent, but this is carefully described in the bar charts section of Chapter 4, Presentation of Results.

These incidence sections are then followed by a similar layout for data on mortality albeit not necessarily obtained for the same populations.

Each site-specific chapter summarises the main geographical variation (oesophageal cancer has a very high incidence in China) and contributory causes (in this case, alcohol and tobacco consumption). The results are discussed region by region (Europe, Asia and Oceania, and Americas) and an overall comment summarises the main findings and its relation to other research.

This is clearly an important source book for research purposes and the individual site-specific chapters made fascinating reading. However, what is most impressive is the careful description of 'methods' given in Chapter 3.

In any research work, and especially those as difficult, time-consuming and comprehensive as that summarised here, it is vital that a clear description of method is provided. Thus the authors describe how they used the statistical techniques of age–period–cohort modelling, age representing the age at death, period the calendar year of death and cohort the year of birth of each individual. They describe the different models which may be used, for example the age–period model in which cohort has no influence on incidence and the age–cohort model in which period has no influence. The extension of each model to describe non-linear changes with, for example, calendar time (period) are also described. A notation (see p. 35) is also introduced to summarise the best model describing the changes illustrated by the graphs. The six steps taken when selecting the best model are described, as are the associated tests of statistical significance and goodness of fit.

The best model, together with the value of the goodness of fit, is reproduced for each registry providing data on a sex and site-specific basis in the tables facing the illustrative panels described above. It is a pity that the explanation provided of goodness of fit in the footnote is somewhat terse. It will not be clear to many readers what a 'normalised likelihood ratio chi-square' is and particularly why it can be negative! The use of D for the drift-model (itself possibly requiring further explanation) is likely to be confused with **D** for deviance.

There is an extensive references section containing 310 references in all. This is, in itself, clearly an important research resource.

There is no doubt that this book will form the basis for much further research. It should be on the bookshelf of those concerned with all aspects of cancer epidemiology and there is a great deal to interest those more concerned with the clinical aspects of cancer medicine. It is a tribute to the high quality of work we now expect from the IARC.

Finally, the authors should be congratulated in collating, analysing and describing so clearly data obtained from 42 different countries.

David Machin